# Climate adaptation plans and “green jobs”: challenges for implementing a responsive, multi-skilled workforce in Worcester, Massachusetts

**DOI:** 10.3389/fsoc.2025.1537311

**Published:** 2025-02-12

**Authors:** Varun Bhat, Sarah Strauss

**Affiliations:** ^1^Community Climate Adaptation, Worcester Polytechnic Institute, Worcester, MA, United States; ^2^Department of Integrative and Global Studies, Worcester Polytechnic Institute, Worcester, MA, United States

**Keywords:** green jobs, green job characteristics, green job types, job analysis, green jobs NLP

## Abstract

Cities across the United States are affected by climate change impacts, and several cities have adopted climate adaptation plans to respond to this growing threat. These plans outline interventions that require a multi-skilled workforce working towards “green” or sustainable goals. However, the “green jobs” linked to these goals are ill-defined and misunderstood among employers and job seekers and can cause gaps in implementing the interventions outlined in the climate adaptation plans. Therefore, it is important to analyze the current state of green jobs in US cities and understand what employers, job seekers, and others think of the green jobs market. We conduct this analysis with the help of natural language processing and qualitative coding in the City of Worcester, Massachusetts, USA using job data from Indeed and semi-structured interviews. We find that the current green job market in Worcester is siloed around green jobs requiring degrees and that non-degree green jobs are significantly less present. Moreover, most green jobs are located far away from Worcester, making them unattractive to job seekers, influencing the overall image of green jobs among job seekers. Finally, both policymakers and employers are unclear about the definition of a “green job” impacting a job seeker’s search behaviors. All this points to a vague description of green jobs and green workforce, that can significantly impact Worcester’s ability to achieve a climate-ready workforce and to achieve its climate adaptation goals.

## 1 Introduction

Cities in the United States are increasingly preparing for climate change by adopting climate adaptation plans (CAPs). These CAPs outline actions such as “installing solar panels or building better houses,” whose outcomes are connected to achieving short and long-term goals in sustainability sectors such as clean energy, transportation and food systems, housing, waste, water, and education. These actions are in response to climate impacts that are ever-changing and unique to a specific city, and that is why they need a workforce that can adapt to new standards and technologies while ensuring that these standards are compatible with current or old systems. The actions outlined in CAPs, therefore, require a relevant and multi-skilled workforce^1^ that can respond to climate events, install new infrastructure, or perform regular maintenance, often in settings involving multi-sector or multi-industry collaborations. This workforce is called a “green workforce” (Pearce and Stilwell, 2008; Walsh, 2008) and the jobs associated with it are called “green jobs.” However, observation and prior research have shown that green jobs and green workforce planning in CAPs often lack nuance. They are narrow in focus, deprioritized, or suffer from a mismatch in top-down expectations to ground-up needs (Kane and Tomer, 2023), creating “implementation gaps” when implementing plans at the ground-level^2^ (Hering, 2018). Therefore, scholarly work needs to analyze CAPs for implementation gaps, and green jobs and workforce are an important part of this analysis. Our pilot study analyses aspects of green work such as what kinds of green jobs are in the market, what kind of training the workforce possesses, and where people are looking for these jobs. With the ubiquity of platforms such as Indeed or LinkedIn, and the emergence of green job-focused platforms such as Green Jobs Network^3^ or Climatebase,^4^ it is necessary to understand the different types of green jobs and who is looking for them. By doing so, we can provide a better picture of who the current audience for the green job market is, who it excludes, and why. In climate change and its related issues, wording and phrasing also influence success in attracting attention to the problem, so much so that Chan and Lin (2022) report, “the way we communicate the effects of climate change needs to be considered.” Similarly, we need answers to questions such as whether adding the word “green” might drive people away from seeking green jobs. All these analyses help provide a clearer idea of what workforce cities need and how to attract them to achieve the goals outlined in their CAPs.

We pioneered this type of analysis in the Commonwealth of Massachusetts, USA, by analyzing the local green jobs in the City of Worcester, MA. We chose this city because it has a well-developed CAP, called the Green Worcester Plan (GWP) consisting of ten distinct goals (Department of Sustainability and Resilience, 2021). The tenth goal focuses on “sustainability education and training for green jobs.” Moreover, the GWP also won two national awards from the American Planning Association (City of Worcester, 2022). Worcester is also a “gateway city”,^5^ which makes it a critical location for achieving climate adaptation success in the New England region. Still, the GWP lacks specificity around the meaning of the “green jobs” goal, and many paths to achieving this goal are still undefined. Thus, our primary questions are–

1. What are the different skills, roles, and responsibilities that a worker needs to have before entering the green workforce in Worcester?
2. What type of workers does the current green job market serve vs. what type of workers does the GWP require?

We looked at 2668 jobs within 50 miles of the city using “clean energy,” “renewable,” “green job,” and “sustainability” as our keywords. We chose these specific keywords because of their for the City of Worcester in achieving its economic and workforce goals in the GWP. With the help of Natural Language Processing (NLP)^6^ techniques like cosine similarity, frequency analysis is a bag-of-words approach we extract key terms from job descriptions that speak to the types of skills demanded, their overlaps, and differences. We then put these jobs onto an interactive map to see how close or far from Worcester they were. This is important because many of the analyzed jobs require significant driving, isolating the populations who do not own a car or cannot drive. We also conducted a qualitative study involving three career professionals in reputable universities within the city to understand what type of graduates are specifically looking for green jobs and whether they understood green work and the skills demanded of them. The career professionals were also shown the interactive map to gauge their reaction on how such a tool could be useful to recent college graduates looking for jobs then. In the following section, we highlight previous examples of how a lack of workforce has affected climate adaptation in US cities while also highlighting the various barriers that workers face today when trying to access green jobs.

## 2 Current state of green workforce in CAPs and their barriers

Some CAPs lack plans for workforce development, while many others are very narrow, with their focus limited to a few sectors. A narrow focus is an issue since cities require multi-skilled individuals who can respond to different climate needs. For example, the Baltimore Metropolitan Council could not work on stormwater management due to a lack of stormwater workforce (Baltimore Metropolitan Council, 2016).^7^ The “required workforce” not only involves operators and technicians but also designers and administrative workers; the entire system needs to be considered. Similarly, regions like Mexico Beach (located in Florida, United States) have reported slowed rebuilding efforts post-Hurricane Michael due to a lack of construction and administrative workers (Young and Miller-Medzon, 2018). In a more recent example, a shortage of experienced lines-people impacted Puerto Rican cities during and after Hurricane Maria (Kunkel, 2020). Thus, a city needs access to a full range of multi-skilled individuals in its workforce to achieve CAP-related goals.

A major barrier to building a multi-skilled, green workforce is the ambiguity of the term, “green job” (Bowen, 2012; Deschenes, 2013; Kozar and Sulich, 2023; Martinson et al., 2010; Mathieu, 2024; Stanef-Puică et al., 2022). This ambiguity can cause a multitude of issues, such as employers not knowing what a green job is, workers being unable to apply for a job that matches their skills, and jobs being misclassified. Not all “green jobs” are good jobs (Stevis, 2012; Uehlein, 2010) and previous research has shown that “the issue is not a shortage of labor or workers to complete climate work but rather the quality of jobs and the lack of investments to expand training, access and equity among these jobs” (Spaans et al., 2024, p. 8). Cities also have custom green job definitions and therefore there is a lack of unification and identity on what the term means (City of Boston, 2019; City of Los Angeles, 2019; Roberts and Yewdall, 2021). This lack of green job identity can affect job seekers since “green job” skills differ in many ways from other jobs (Consoli et al., 2016). For example, the Interstate Renewable Energy Council (IREC, 2024, p. 30) reports that it is important to learn not only technical skills but also whether a person is comfortable climbing heights (such as roofs) to perform their green job as an installer, which may not be a part of the job descriptions (which we also highlight in our analysis later). Moreover, because many green jobs are identified primarily as white-collar, energy-related, or gated to degree-holder applicants, some of the workforce can become alienated, and not seek to participate in the green economy. For example, Curtis et al. (2024, p. 3) found, after studying 300 million jobs, that most of the rise in green jobs could be attributed to “EV-related jobs.” Similarly, Denham et al. (2024) found that the adaptation job market in Australia was specifically focused on “consulting, banking, and finance roles” indicating that “adaptation is for those who can afford it.” Kane and Tomer (2023) found that “40 of 50” cities in their study “emphasize energy projects when discussing workforce needs, but considerably fewer cities emphasize workforce needs in terms of buildings, transportation, or other parts of the built environment.” We also found a similar pattern for the O*NET “Green New and Emerging Occupations” where 27 out of the 92 listed occupations were for engineers; most of them were energy focused such as fuel cells, biofuels, solar, hydroelectric, as well as a few robotic-based jobs. Additionally, with the existing political divide in the United States around climate change (Dimock and Wike, 2020; Knuth, 2020), the term “green” in green jobs could also act as a barrier that drives skilled workers away from them because of differing political beliefs or a desire to stay away from politically charged terms. Conversely, as more and more people concerned about climate change enter the workforce, they tend to look for jobs that contribute positively to the environment and the climate. Companies are also incorporating socially conscious thinking into their policies and practices (Klingenberg and Kochanowski, 2015), and reports have shown that companies and businesses need to have “green jobs” and sustainability thinking (Deloitte, 2024; Khosla, 2025) and therefore an ambiguous definition can be a barrier to hiring. For example, a former department head in Civil, Environmental and Architectural Engineering at Worcester Polytechnic Institute, commented that “employers say that they are having to respond to the fact that graduating students, and recent alumni, are strongly interested in jobs with a sustainability or climate resilience dimension. From this viewpoint, both employers and job seekers care about the values and labeling of ‘green jobs’” (CM Eggleston, personal communication, January, 17, 2024). In their survey of 1,306 job seekers, Cutter et al. (2021, p. 21) find that 29 percent expressed that they “would like to have a job that helps to reduce or halt climate.” Thus, the term “green jobs” and the narrow focus of its definitions all contribute to the barrier that prevents us from achieving a multi-skilled workforce in our cities.

Another barrier is the fact that climate change acts as an intensifier of existing problems. Events like hurricanes, floods, erosion, and sea level rise (World Wildlife Fund for Nature, 2024) demand that our built environment and infrastructure withstand even more stress. For example, a city’s energy demands could increase due to an influx of climate migrants, leading to a higher demand for energy workers than initially planned. In such cases, the city government would now need to consider which workforce to retain for energy systems installation, operations, and maintenance and which workforce to displace to other departments like water, sanitation, transportation, and healthcare. Moreover, cities would also need to update their codes, standards, and practices to have the capacity to address these events effectively. However, by the time the workforce gets trained, there is a good chance that the situation has already worsened, rendering the new standards obsolete. This delay, where city governments need to play catch-up can cause emergency situations. For example, in 2023, two dams in Leominster, MA were dangerously damaged, due to the unprecedented flooding causing them to nearly fail (Casey, 2023; Casey and McCormack, 2023). Therefore, when the non-uniformity of green job definitions and the political divide around climate change is added to this dimension of climate intensification effects, it could lead to additional delays in long-term and short-term city planning efforts over time. Thus, as the climate continuously changes, cities need to get better at defining workforce requirements and ensuring that the workforce is trained and ready to address changing needs.

Finally, many resources, skills, and infrastructural barriers affect a person’s job access. For example, factors such as access to electricity or a computer, language barriers, document mismatches caused by systemic delays, housing, or distance from work could hamper people from getting work (Kanas and Kosyakova, 2023; Lim et al., 2023). Companies also have trouble understanding what climate adaptation work looks like and that causes them to misclassify jobs on online platforms. For example, Szabó et al. (2023, p. 407) found that “a large proportion of leading employers do not or hardly communicate their company’s efforts towards environmental sustainability, especially in their advertisements.” There are also certification barriers where a person may have the skill but lack the certification required by the local law or a green job (Hooper and Huang, 2024; Hughes, 2018, p. 48–49). For example, the State of California could face a potential slowdown in rooftop solar installation due to the job being limited to C-10 electrical contractor license holders, effectively shutting out people who already have the skill or experience, but not the certificate (St. John, 2024). Finally, with many jobs being advertised and applied for online (Choi, 2023; Faberman et al., 2022; Kroft and Pope, 2014), people without digital skills or access to a computer could also be impacted (Wheeler and Dillahunt, 2018).

Scholarly work around the subject of green jobs and access has suggested that planners must look at jobs from a multi-perspective view involving various societal, historical, geographical, and cultural factors. By incorporating this view into their CAPs (Bierbaum et al., 2013; Hamin et al., 2014; Radonic and Zuniga-Teran, 2023), planners can also adopt a Just Transitions framework ensuring that future workforce and greening policies are fair for all and embody a bottom-up approach (Cannon et al., 2023; Chu and Cannon, 2021; Fiack et al., 2021; Malloy and Ashcraft, 2020). This framework is a departure from a trend of top-down, exclusionary planning and a journey towards an inclusive environment that fosters skills training, and contextualized development at the local level (International Labor Organization, 2015; Malloy and Ashcraft, 2020). *We therefore theorized that a good place to start would be to analyze the job market by uncovering factors such as green job location, roles, responsibilities, and other barriers of entry, identifying which economic class is served and who is marginalized, and whether the job seeker can access meaningful and well-paid, green jobs through existing job platforms.* Our study includes data science techniques for quantitative data, backed by findings from semi-structured interviews to identify the current state of the green job market in Worcester. In relation to the needs of the CAPs, there will never be a perfect fit between jobs offered and skills in demand, because climate change will constantly shift industrial standards and short-term and long-term needs. However, mapping the skills in demand, jobs offered, and mismatch in skills vs. CAP needs in job ads will help provide insights around how green jobs are presented in the market and what is missing when it comes to providing better fit for job seekers. By examining the relationship between these factors and what the GWP has identified as needs, we can help close the gap between what green jobs mean to employers and what the city has identified as GWP needs, thereby increasing the ability of workers to find quality green jobs.

## 3 Materials and methods

Our goal here was to understand the positions, skills, and responsibilities of local “green jobs” in the City of Worcester. To do this, we looked at job advertisements on job platforms that are popular mediums for advertising and applying for green jobs (Kroft and Pope, 2014; Faberman et al., 2022; Choi, 2023). We chose job platforms as we are already seeing a push where platforms are trying to make the job search process more inclusionary by adding different tools and knowledge articles to make it easier for the job seeker. For example, Green Jobs Network has automated cover letter generators and programs that give sample interview preparation questions.^8^ Moreover, the platform also places a special emphasis on jobs and companies that have a history of positively impacting communities through spotlighting and newsletters.

We collected our data from *Indeed.com* since we wanted to focus on a platform that included a more generalized job market. Platforms like the Green Jobs Network are focused only on green jobs, which would attract a self-selecting audience, while we wanted to focus on a wider set of jobs and job seekers. We used a method called scraping^9^ while ensuring that we did not infringe on the site functionality. Scraping is an indirect, automated method to collect large amounts of data from websites (Khder, 2021). A direct way to collect this data is through Indeed’s native Jobs API program, that has not had a way to obtain direct job data for quite some time. To scrape this data, we decided to use software called ParseHub.^10^ It has robust features such as exporting to Excel CSV and controlling what data to extract and what not to. The user interface is also easy to use making it easy to scrape and collect the required data. We ran the software for a week during late hours to not affect the website’s performance. We also limited the number of requests the program sent per minute to minimize our impact on the site’s traffic. We created different sessions of data extraction governed by our keywords—“clean energy,” “renewable,” “green job,” and “sustainability.” We chose these keywords due to their significance for the City of Worcester. For example, “clean energy” has been identified as one of the primary industries which will see a growth in new occupations (City of Worcester, 2021, p. 30) post-implementation of the GWP. The city is also planning to evaluate “economic analysis of sustainability and resilience projects … for costs and benefits on a life cycle basis.” (p. 30). Similarly, Goal 10 of the GWP aims to make “Worcester the regional center for sustainability education and training for green jobs.” (p. 12). By analyzing these keywords, we also aimed to cover two out of the five “big picture” visions for the city (Energy and Education and Awareness),^11^ thereby making our results relevant to the city’s long-term planning goals. We collected 2,668 green jobs within 50 miles of Worcester, which as per the U.S. Census Bureau is the limit to which Americans are willing to drive for work (Rapino and Fields, 2013). Of those jobs, 697 are “clean energy,” 686 are “green,” 484 are “renewable,” and 801 are “sustainability” jobs.

**Figure 1:**
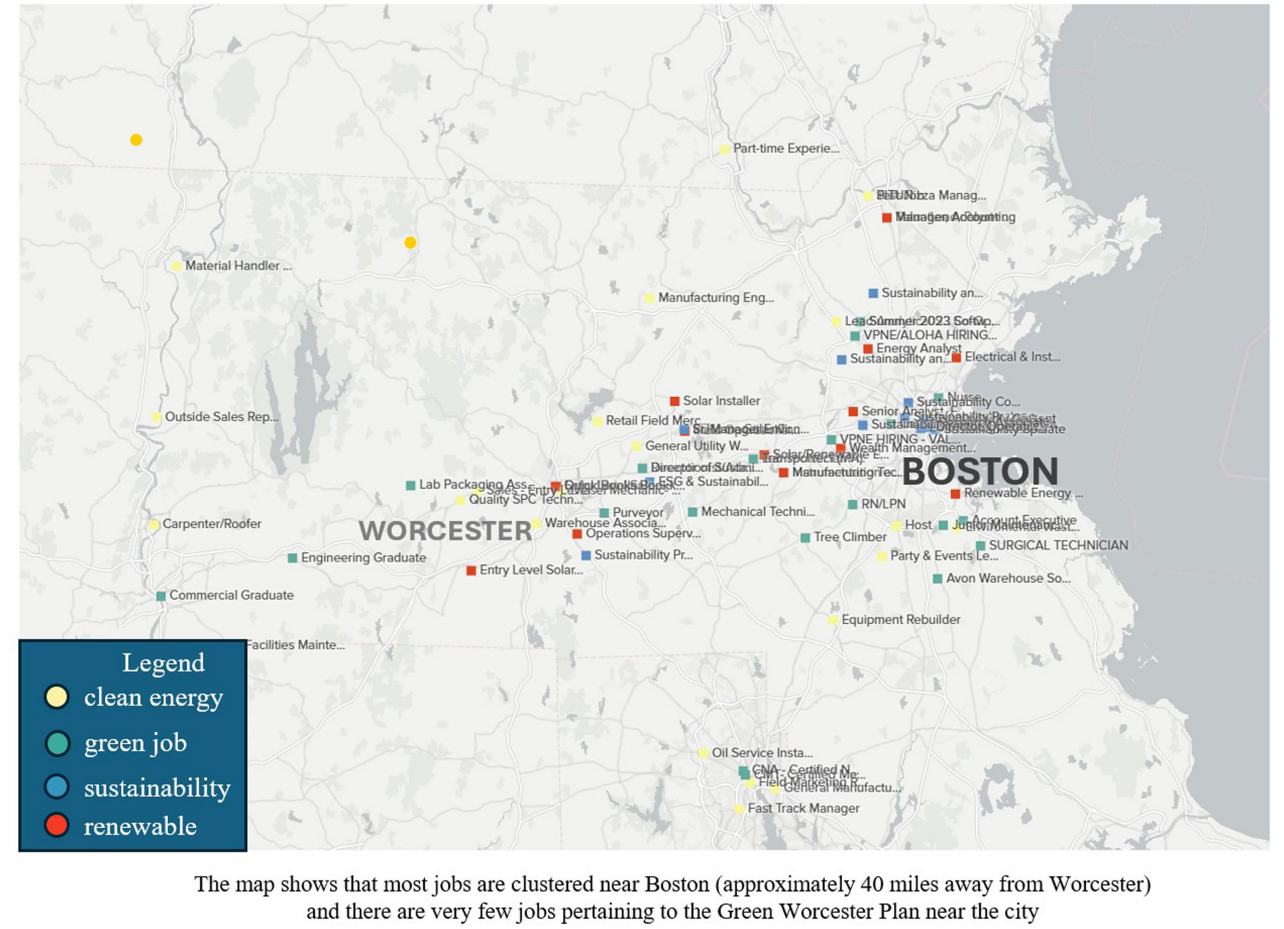
Map showing a few of the analyzed jobs near Worcester.

After collecting the job data, we conducted three semi-structured interviews over Zoom. The participants worked as career development professionals in Worcester-based universities. All participants were given an oral consent statement as per the study protocol and we used Zoom’s recording feature to record the audio. As a part of the interview, we also output some jobs from the collected data onto a map using a tool called Kumu.io^12^ (see Figure 1). The goal was to understand the career professionals’ thoughts about using digital technologies to understand green jobs. We also wanted to represent the jobs on a geospatial map showing how close or far the jobs were with respect to the City of Worcester, Massachusetts, USA. Due to time constraints, we could only showcase 50 jobs from each dataset (*N* = 200). Each job had a Job Location (sometimes with full or partial addresses, or just city names) that Kumu picked from the spreadsheet and input into its mapping software. Our interview prompts had four topic areas—(A) Questions about field of work, (B) Questions on the knowledge of the Green Worcester Plan, (C) Questions on campus sustainability, (D) Thoughts on the Kumu.io tool. Each interview ran for 60 min maximum, and all responses were in English.

## 4 Findings from collected data

Since job text is typically written by a person, natural language processing methods are useful (Granata and Posadas, 2024; Paliński et al., 2024; Ronran and Pairot, 2024; Schultheiss et al., 2023). We used the Python language due to its extensive repository of natural language and machine learning libraries that were a good fit for our text-based datasets. We used the pandas software^13^ in conjunction with Natural Language Toolkit (nltk),^14^ a word cloud generator, and sci-kit learn (sklearn)^15^ for data analysis. Using these tools, we pre-processed our data to automatically remove unnecessary words and phrases to analyze the different job types and job descriptions. Through the analysis, we uncovered important green job skills and investigate the characteristics of jobs across different sectors and seniority-levels.

We also auto transcribed our interview data using a tool called *Otter.ai*.^16^ Upon transcription, we cleaned the transcript to be grammatically accurate before performing inductive coding to identify the various themes that emerged from each interview. We have noted four distinct themes, namely, future of green jobs, green job titles, green jobs knowledge (or awareness), student knowledge about sustainability. These themes highlight crucial aspects of green jobs that have been implemented well in society, while also shedding light on what more can be done in Worcester.

### 4.1 Pre-processing

**Figure 2:**
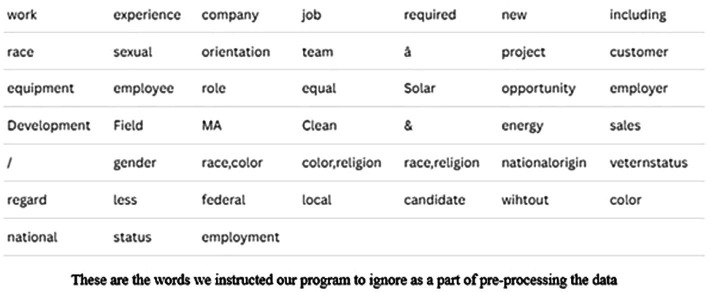
Snapshot of words ignored in data pre-processing.

Scraping is not a precise extraction method and therefore our data had several special characters, unidentified symbols, and common English words. So, after collecting 2,668 jobs from four different keywords, we needed to do some pre-processing. For example, we needed to remove words related to the equal opportunity employer statements for this analysis. Therefore, we developed a dictionary of words that we programmed python to ignore using nltk’s stopwords corpus. Figure 2 shows a snapshot of the words we asked our program to ignore. After pre-processing the data, we extracted the data into four datasets within Excel, using four different sheets. We did this to make it easier for us to analyze the data. Each dataset correlated to one of the keywords, “clean energy,” “renewable,” “sustainability,” and “green job.” Each dataset has six columns, namely, (1) Job Name, (2) URL, (3) Job Title, (4) Job Location, (5) Job Type, and (6) Job Description.

### 4.2 Word frequency in job titles and responsibilities

For our first series of analyses, we did a frequency count of the various job titles and responsibilities for each dataset with the help of bigrams and unigrams. Bigrams (or 2-grams) are tokens that contain two words together (for example, “solar-installer” or “sewage-technician”). We needed to use bigrams since doing a frequency analysis of single words (or unigrams) is bound to miss some context. For example, there could be two different jobs titled “solar installer” and “pipe installer.” A frequency analysis of the unigrams (solar, installer, pipe, installer) would return [solar = 1, pipe = 1, installer = 2]. This would hamper our analysis since it is also important to understand from which sectors the installer jobs originate.

**Figure 3:**
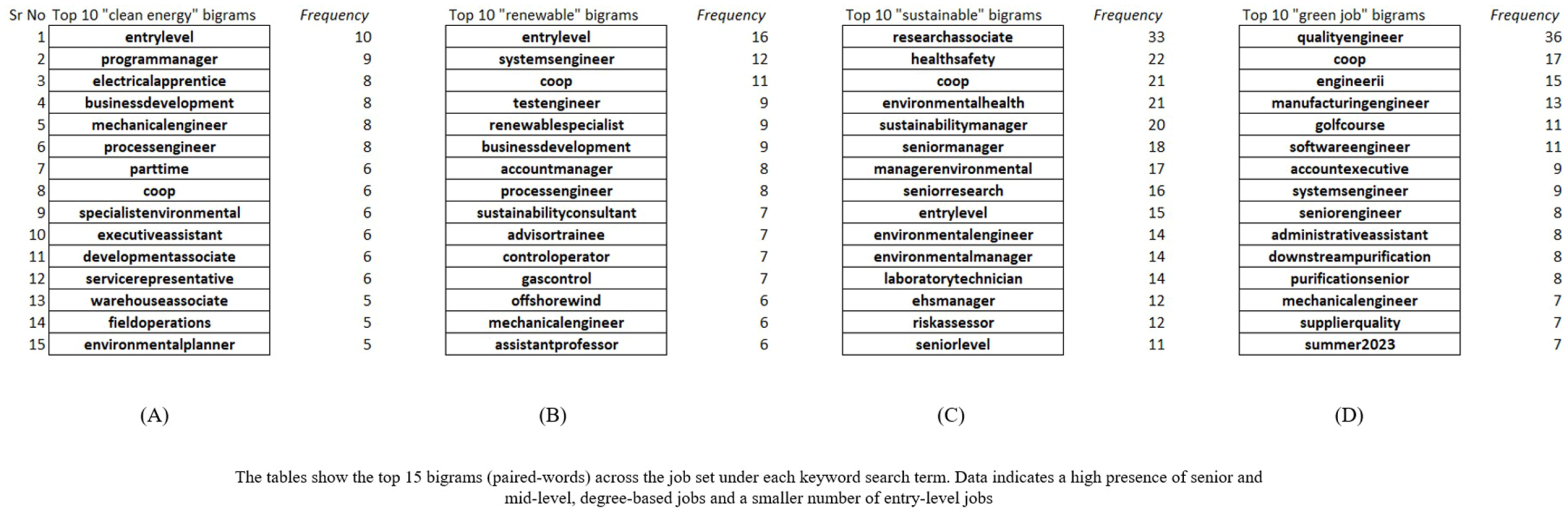
Frequency of job title bigrams from each keyword. **(A)** “clean energy” bigrams, **(B)** “renewable” bigrams, **(C)** “sustainable” bigrams, **(D)** “green job” bigrams.

**Figure 4:**
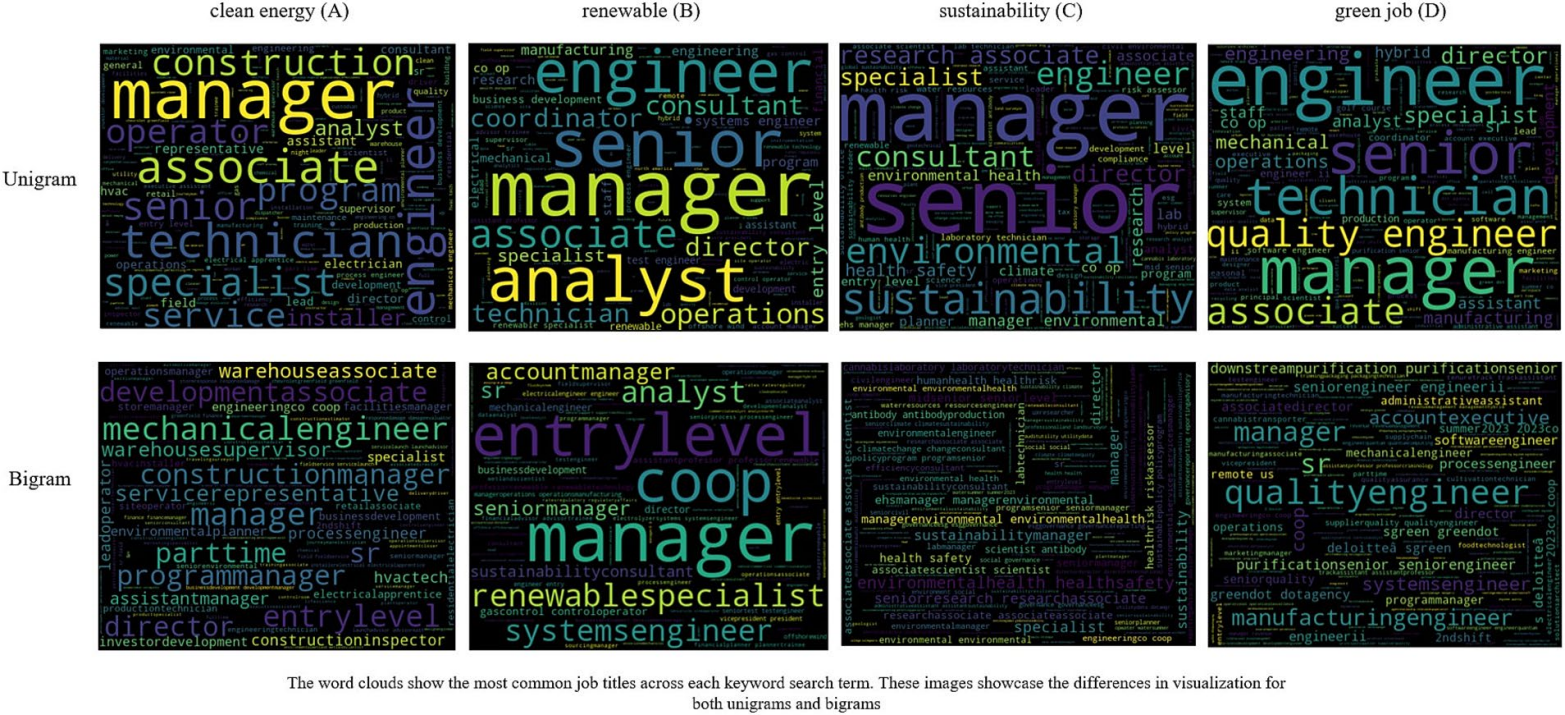
Wordclouds of job titles from each keyword. **(A)** “clean energy”, **(B)** “renewable”, **(C)** “sustainability”, **(D)** “green job” clouds.

We created four arrays of only Job Titles from the original datasets. Similarly, we created another set of four arrays for Job Descriptions. We did not use Job Name here since it often contained the same data as Job Title (most likely due to employers duplicating data entries for ease). Next, we stripped the arrays of punctuation marks, whitespace, and applied the nltk stopword corpus to remove the unnecessary words. The resulting arrays contained words related to our analysis. Finally, we created the bigrams by shifting the array by one index and grouping the words. We then counted the resulting groups of Job Titles and Job Descriptions for the clean energy, renewable, sustainable, and green job datasets using pandas’ *value_counts()* method which returned the frequency count. Using the frequency count, we created a list of the top 15 bigrams in each dataset which we highlight in Figure 3. We also created a word cloud of the most frequent job title unigrams and bigrams in each set which shows high frequency words in large-sized fonts (see Figure 4).

We found that many jobs were at senior or management level across all four keywords. Several other jobs were mid-to-senior level with “associate” and “engineer”-level jobs being most frequent Although the bigram “entry-level” appears as one of the top 15 bigrams (see Figures 3A,B), entry level positions such as “technician” and “installer” were few and far between. After analyzing the datasets further, we also found that most of the listed jobs were full-time in nature. Part-time, remote, or contract-based work was also less. We also found that only a few job descriptions included information about the job type.

Moreover, words such as “leader” or “leadership” were more common in the job descriptions of mid-and senior-level jobs and very uncommon in the entry-level descriptions, which is important, since it could lead to a lessened upward mobility among entry-level job holders. Energy companies like Revision Energy are already offering their apprentice electricians “job advancement opportunities through leadership skills development and mentorship.” (Revision Energy, n.d.). Therefore, Worcester and the GWP must consider this viewpoint from a workforce development aspect for the future of “green jobs” and “green work.” “Microsoft Office/Microsoft Suite” was a common bigram found in the job descriptions. This finding is important from an educational training perspective, where course curriculums can place emphasize digital skill training for “green jobs.”

The job set also indicated the analyzed jobs have higher technical skill requirement indicating a need for upskilling, certification, or university degrees, because of the high frequency of bigrams in the job descriptions, such as “hydrogen electrolysis,” “electrolytic systems,” and “hydrogen experts.” This is also characterized by the low frequency of words like “control wiring,” “boilers, pumps” and “longest-tenured operator.” We also found that words that indicate physical work such as “lifting,” “pulling,” or “standing,” which generally are a common requirement in entry-level jobs, were very low frequency. The analysis of the job descriptions also highlights a few potential implementation gaps in the GWP. For example, in the case of vocational training, the city aims to “establish training programs at the Worcester Technical High School for sustainable building systems and renewable energy.” (p. 72). It also makes similar commitments to find educational and non-profit partnerships with other vocational schools and academies for “Workforce and Youth Workforce Development for maintenance of green infrastructure and biodiversity/pollinator friendly landscapes” (Department of Sustainability and Resilience, 2023, p. 24). Yet, there is no mention of a training plan or pipeline for skills such as Microsoft Office, leadership, or physical fitness, which, as our job description analysis shows, is important for the job seeker to possess. Acquisition of digital skills could also involve additional costs to the city (software licensing, purchasing machines, hiring instructors) and not having a concrete pipeline or plan would cause implementation gaps in the workforce development plans.

### 4.3 Similarities between job descriptions of different job titles

**Figure 5:**
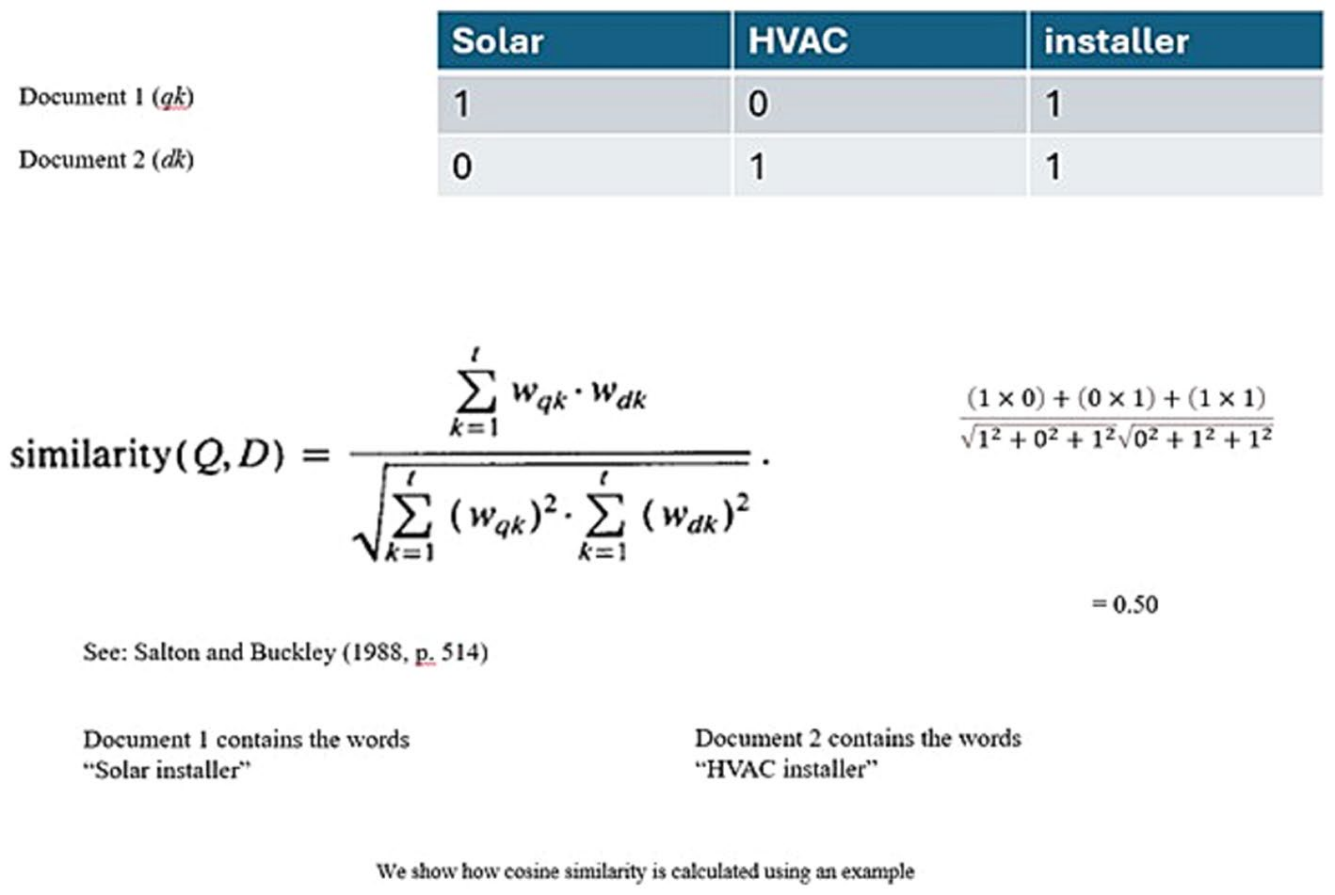
Example illustration of cosine similarity calculation - see Salton and Buckley (1988, p. 514) for equation.

We also analyzed how similar the job descriptions from each dataset were using cosine similarity, which calculates the similarity score between two documents by recursively taking the cosθ between a set of individual vectors/terms. For example, if two documents contain the terms “solar installer” and “HVAC installer,” then the similarity score would be 0.50 (see Figure 5 for calculation).

**Figure 6:**
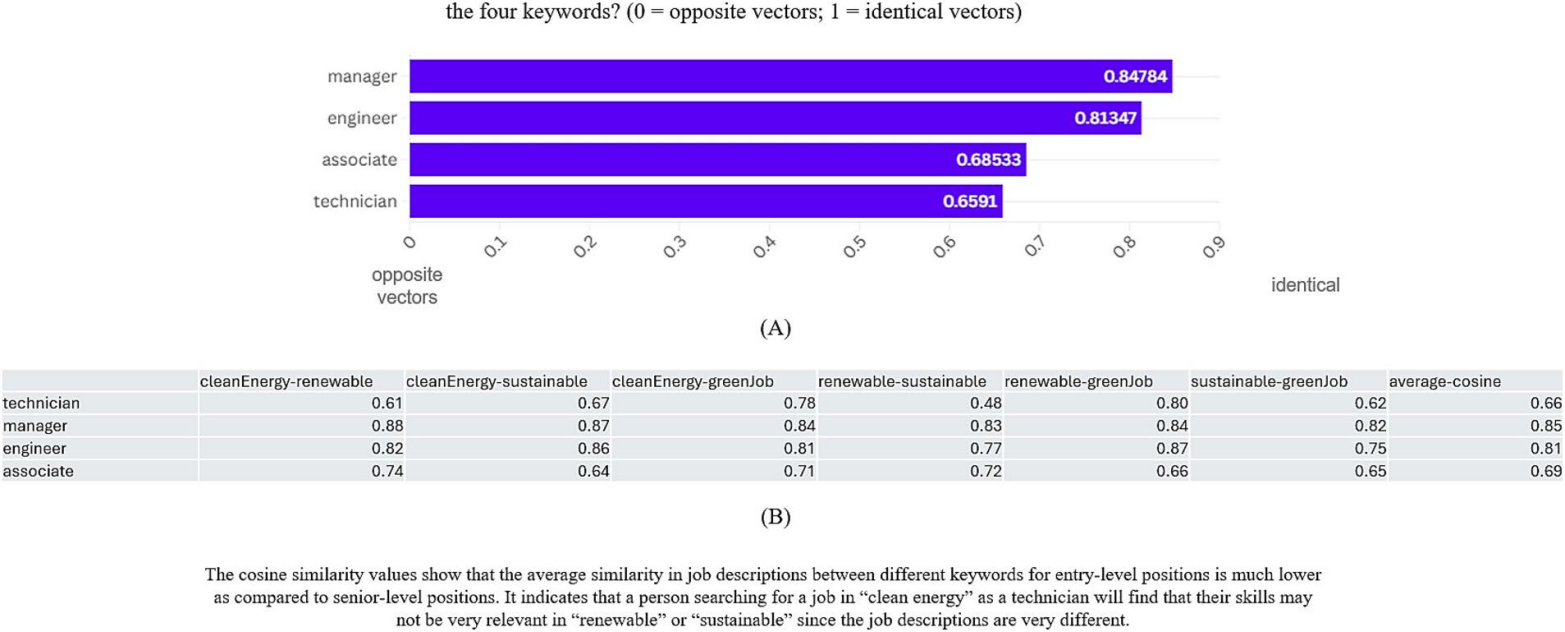
How similar are the job descriptions within each role across the four keywords? (0 = opposite vectors; 1 = identical vectors).

We programmed our Python application to use the inbuilt cosine similarity calculator in the nltk toolset across our four documents, i.e., “clean energy,” “renewable,” “sustainability,” and “green job.” We eliminated keywords and phrases such as “ideal candidate” and equal opportunity statements that did not directly relate to our analyses. Due to the large number of jobs in our dataset, an analysis of the similarity in job descriptions across all jobs was not feasible due to time constraints. Therefore, we looked at the similarities in job descriptions for “technician,” “manager,” “engineer,” and “associate” roles, which we found covered different job levels (senior, mid-level, entry-level), job types (full-time, part-time), and barriers of entry (degree-holding, management training, GED or diploma). The program compared the documents in a round-robin approach to generate cosine similarity scores between two document pairs for each role. For example, for the “manager” role, the document pairs would be clean energy-renewable, renewable-sustainability, sustainability-green job, and green job-clean energy. Finally, we averaged the scores across the four document pairs to generate an averaged similarity score for each role (see Figure 6).

Unsurprisingly, we found that the senior or mid-level jobs, or jobs with high barriers of entry such as managers or engineers, were more similar in their job descriptions when compared across all the sectors. As explained before, we removed extraneous words from the analysis set, and therefore most of the words we look at relate to skills, roles, responsibilities, and expectations from the employee. The homogeneity indicates that people working in the high barrier of entry roles are more employable regardless of how the employer markets their job. However, people at the technician or associate level may not possess easily transferable skills or experiences from their old jobs and may not have the desirable employability as compared to their peers.

### 4.4 Looking at various job types

**Figure 7:**
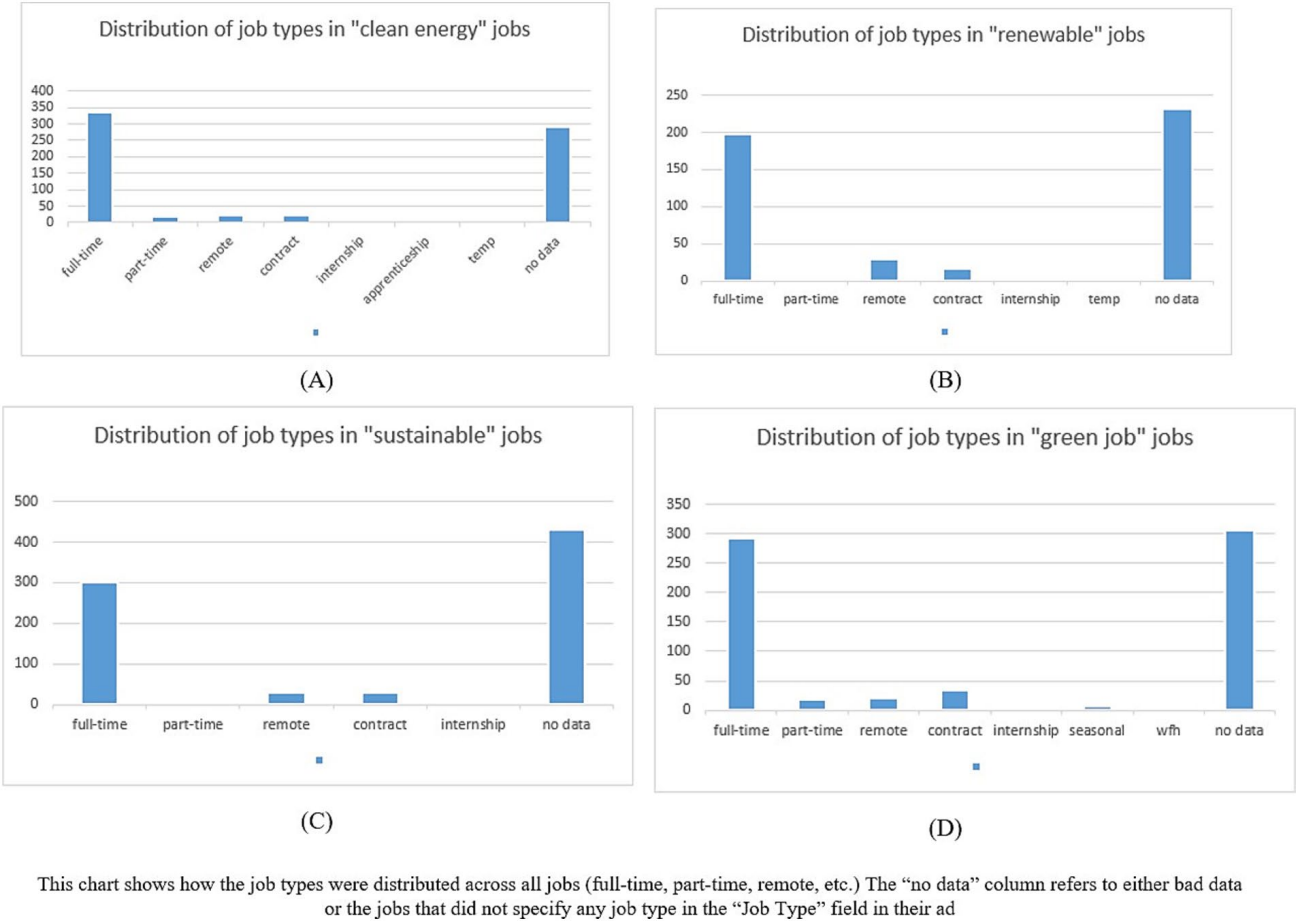
Job distribution according to job type.

We also noticed many full-time jobs as opposed to part-time or contract-based work. It is also interesting to note that many jobs did not mention the type of job explicitly and could cause many issues for the job seeker looking for a green job (see Figure 7).

### 4.5 Policy implications: barriers distilled from perspectives by Worcester career services professionals

**Figure 8:**
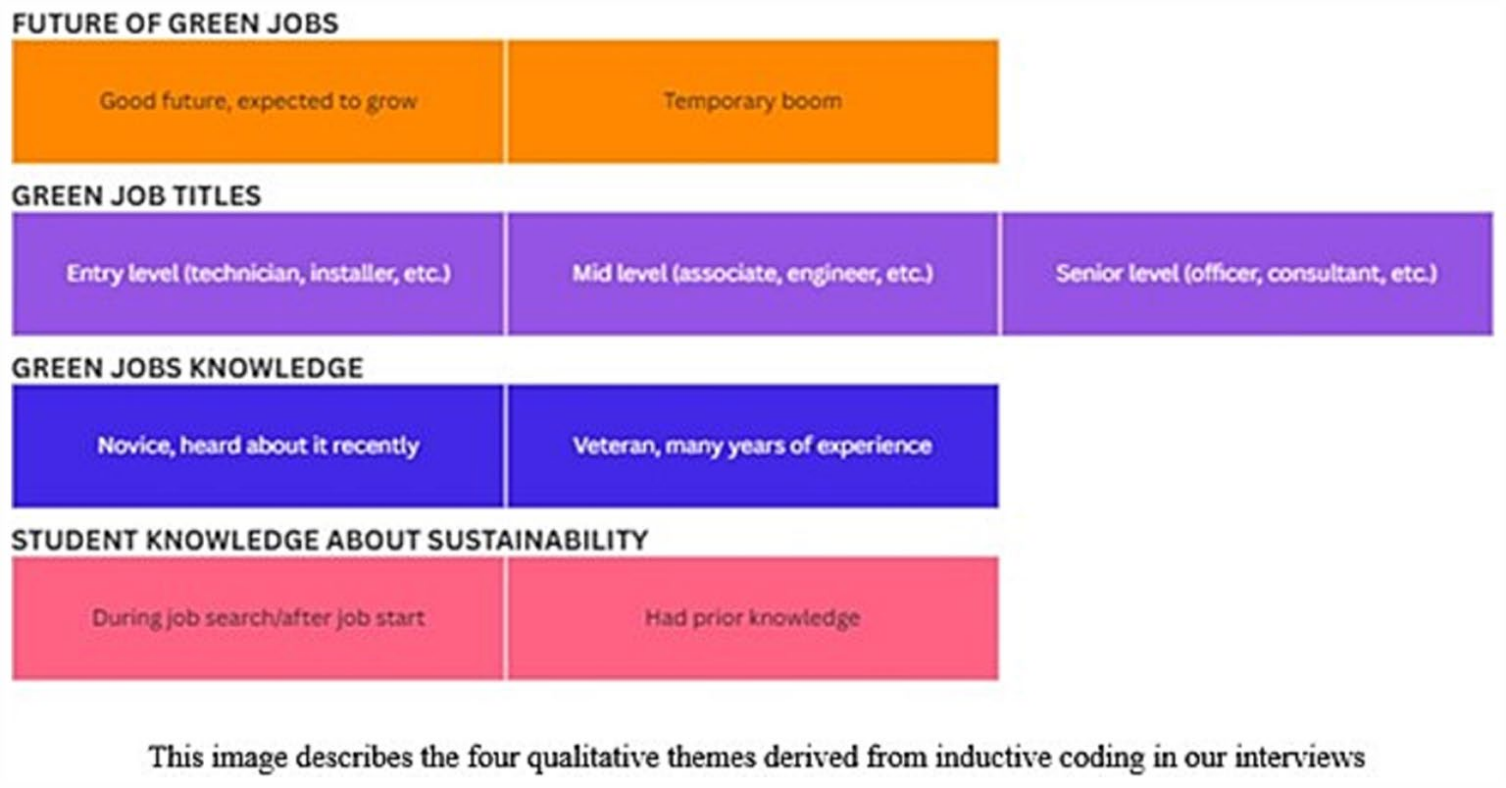
Inductive codes from interviews.

We performed inductive coding of the interviews and found four distinct themes and nine sub-themes. The themes and their description are shown in Figure 8. Our qualitative study also had conclusions that reinforced our quantitative data analysis. Although we identified multiple themes, we only display the results related to our main questions, which are:

1. What are the different skills, roles, and responsibilities that a worker needs to have before entering the green workforce in Worcester?
2. What type of workers does the current green job market serve vs. what type of workers does the GWP require?

When asked about what they found the top three green or sustainable jobs in the market to be, all responded with mixtures of officer, consultant, or specialist-type roles, often requiring a degree. One respondent said, “we are not … stumbling upon entry level positions that have a green component mixed into it.” They also went on to add that there was something “still missing” in terms of advertising their green jobs. They added that it was “almost as like a marketing campaign … but … not truly a focus of their mission.” Another respondent said in an example of the civil industry that, “for construction companies, there’s a lot of work being done for … LEED buildings, and they do not always market themselves this way.” The same respondent also said in terms of bad or missing job data that, “people are being very specific in their desires for work … remote, hybrid, that type of thing. And sometimes it’s just that the company … corporate headquarters … says it’s there, but it’s actually not really there.”

When speaking about the City of Worcester’s Green Worcester Plan, all three respondents highlighted that we need more awareness around green jobs with employers and that we need more education-to-job pipelines. For example, one interviewee mentioned it was necessary “to have shadowing opportunities as early as after their first year of college … almost like that pipeline of opportunities … keeping them informed.” In a different example, a career expert said that we need to partner with businesses and employers to increase the visibility of green jobs. On asking “do companies understand what sustainable and green jobs are,” the expert replied “I think it really varies… there are particular people at those organizations that know a lot about that … but I think that for the most part, even people that work at those organizations do not know … So, for example … for construction companies, there’s a lot of work being done for … LEED buildings, and they do not always market themselves that way.” They also went on to say that “the city could partner with organizations to say … let us work together to meet these goals and provide them with some kind of direction … and also on the student and … a person … when people understand the impact they an make … the city of Worcester could come up with something saying … one person in this role makes an impact, and it impacts … in all these different ways for the city as a whole.” In other words, companies are not very clear around the purpose of labeling something as “green” and could use the guidance on how to make their “green jobs” more visible. This concrete vision of how green jobs contribute to society would increase the visibility of green jobs while also clarifying to workers that the skills they possess will ultimately be used for progressing sustainability goals within the city. This type of messaging is crucial to attract skilled workers who are also interested in contributing to plans like the GWP. For example, Jones et al. (2016, p. 7–9) show how recruitment messages around climate change has both a positive and negative impact. The controlled study revealed that emotions of pride, ecological awareness, and belonging due to fitness with personal values are very important signals (p. 7–8) influencing employer attractiveness, and that two-thirds of their participants “reported they were more attracted to the employer as a result of its CI [Community Involvement] or ES [Environmentally Sustainable practices]” (p. 14). The messaging should also not be limited to CAPs but also to job platforms. A standard platform has a limited set of transactions that a job seeker can perform. The job seeker’s interaction with the platform terminates when the seeker has submitted their applications. However, platforms like the Green Jobs Network go beyond the transactional models and are more useful than a standard job platform’s method (like Indeed). For example, Green Jobs Network provides more information on what a green job is, companies that support them, what are the different industries for green jobs, and other automated tools for resume building.^17^ This type of framing and messaging around green jobs on platforms and CAPs invites employers and policymakers to broaden what they think of when they say, “green jobs,” thereby breaking the “business as usual” mindset. It also teaches job seekers to expand their understanding, use different search terms, and explore the different ways their current skillsets can apply to green jobs. Such an approach paves the way for a type of transformative adaptation that “requires a willingness to undertake major psychological adjustments away from what has been normal” (Arkbound Foundation, 2021, p. 82). Similarly, Silvius and Mackinnon (2012, p. 26) note how “it is wrong to assume that multi-barriered individuals will simply take a job based on the job’s availability. Individuals are more likely to pursue training when they deem both the training and prospective jobs to be in their interests.” One of the interviewees also noted that “there is a core mass of students … focused on looking for green, sustainable, environmentally friendly, aware positions.” They also went on to note that they are not, however, coming “upon entry level positions that have a green component mixed into it” (see Table 1 for a summary of perspectives provided by the Career Service Professionals).

**Table 1:** Overview of recommendations from career service professionals.

Themes	Recommendations
Visibility of GWP	City needs to define “what a sustainable education is, what a green job is”. Transference of skills from sectors to sustainability needs to be clearly defined.
Policy/program	Adding internships or shadowing programs for first/s year college students.
Job platform/green jobs	Jobs that are green are not classified as such and vice versa. Showing coursework nearby that can help job seekers get required skills would be useful. Employers need to define sustainability/green component of their job more clearly. City needs to communicate green job resources to universities and employers (for example, which platforms to use).
Other	City could make clear by tracing how exactly a green job role makes societal impact. More collaborations with organizations, especially companies to provide them with direction around green jobs.

This vagueness around green job roles, responsibilities, and career pathways could have real implications on policy planning for climate change responses. For example, due to an unclear understanding of green jobs skills combined with the resulting skill shortage, workers will need “extra time … for on-the-job training and for remedial work, which will lead to delays in delivering products and projects.” (Jagger et al., 2013). Moreover, as certification and standards requirements change at the local or state level (as illustrated in the California solar installation slowdown example on p. 5), the workers might later need to get re-certified, causing both a short-term and long-term shortage in qualified workers. The training and re-certification would also incur monetary costs to the employer organization or the government, and could be met with reluctance, thereby leading to more delays and quality-issues. Moreover, if the cost of upskilling ends up being shifted to the individual (for example, if a person is a contractor or is self-employed), then it might lead to slower transitions depending on people’s willingness to train, take time off work, lack of understanding of its importance, and involved costs. Thus, the GWP and the city need to explicitly clarify what a green job is, how to get a green job from entry, mid, and senior-level perspectives, and what are its contributions to the city’s overarching goals.

### 4.6 Insights from the maps and job locations

Upon showing the Kumu map tool output to the career professionals, all agreed that being able to look at a green job’s location as well as being able to filter it by search keywords or sectors would be very useful for spreading awareness about green jobs to upcoming graduates. One person noted that “job titles are … hard … who might not know … I’m really interested in sustainability, but I’m not really sure … what types of jobs I can look for. Also, I’m not sure what skills they are looking for, if I have the skills that are needed. Similarly, another person noted that job searchers might find it useful to know “what … business also kind of has this green component … your typical high school senior is just unaware of that that could be a potential career path for them.” However, current job tools and platforms are not very conducive to getting this information easily. For example, a recent job advertisement from *Indeed.com*^18^ for a Journeyman Electrician did not mention green jobs or sustainability in the job description and there is little sense of how far the job is from Worcester, contributing to the difficulty of job searching in the green sector.^19^

**Figure 9:**
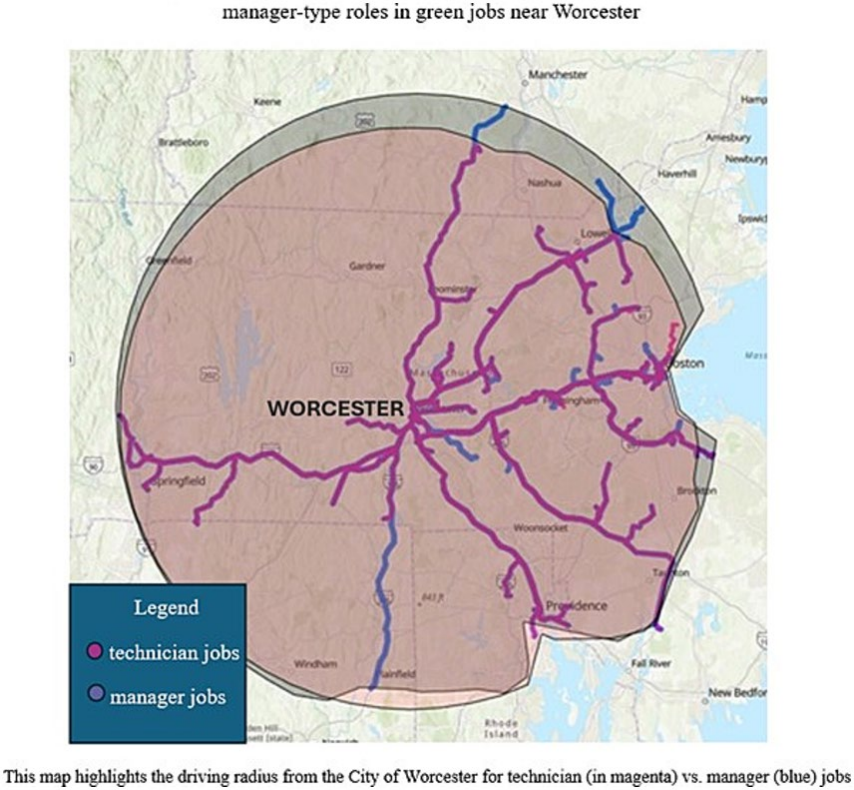
Radial map visualizing driving radius of technician and manager-type roles in green jobs near Worcester.

Moreover, all the green jobs we analyzed in our dataset were quite far from Worcester. We computed the average driving distance between Worcester and each “technician” job (*N* = 115), and each “manager” job (*N* = 170) based on the job titles of our collected jobs (after discarding jobs that did not have a specified location). We used ArcGIS to compute since it also calculates geographical data accounting for the different roads and interstates one would need to take to get to the various locations. We found that a technician would have to drive an average of 36.3 miles to get to the place of work with an estimated average driving time of 43.78 min. A manager would have to drive an average of 39.28 miles with an estimated average driving time of 46.42 min. We also found that many jobs were not located within Worcester and were in other Massachusetts cities such as Marlborough or Boston. Figure 9 shows a radius of where the “manager” (in blue) and the “technician” (in magenta) jobs are located from Worcester (in the center) on an ArcGIS map we created. As seen in the map, the jobs are clustered more towards the east, near population-dense centers like Boston, making the jobs more Boston-centric than Worcester-centric.

Driving distance is very important when it comes to job searches and green job workers are not different in that regard. As noted by one of our interviewees, “we do also have a lot of students that are geographically inclined, and that is … the number one filter when they are searching that they are looking at Worcester … Boston … they are looking at heading back home to Malden or Marlborough … as we are very regional.” In addition to the driving distance, it is also important to note that the public transport system does not extend its network all the way to Boston. There are trains but with the ongoing railway construction and the lack of an express train service, the Worcester-Boston commute is nearly untenable.^20^ This is an issue because as per the 5-Year Estimates from the American Community Survey, around 16 percent of occupied housing units do not have a vehicle^21^ as compared to the national average of 8.3 percent.^22^ A large percentage of workers might need to carpool or find other arrangements to commute to work which could be a major contributing factor to the attractiveness of green jobs far away from Worcester. Moreover, 19.8 percent of persons in Worcester are in poverty^23^ which could affect car ownership or other capacities such as paying for gas costs, tolls, and vehicular maintenance, leading to adverse impacts on a person’s employment. Finally, it also raises a fundamental problem that most of the green, renewable, energy, or sustainability jobs are located *outside* Worcester city or county. It makes them unrelated to the Green Worcester Plan because their benefits would land in other cities such as Boston, thereby not contributing to the actions outlined in the GWP. They also end up draining skills and talents away from Worcester. Therefore, it is critical that the city invest in jobs closer to home to allow broader workforce inclusion and to ensure equal employment opportunities for all.

## 5 Contributions to the field and future work

This pilot study contributes to an understanding of the skills and roles that green jobs currently demand by looking at online job platforms. However, this understanding is incomplete. First, it only looks at one job platform,^24^ and the people who look for jobs there are not necessarily the same people who look for jobs on specialized platforms like Climatebase or at similar but separate platforms like Monster Jobs. As discussed above, in aspects related to climate change, wording and phrasing is extremely important due to the political divide around the issue in the United States (Heikkinen, 2015; Tabuchi, 2017). Skilled workers may not be looking for green jobs on Climatebase or Green Jobs Network due to their differing political views, and it is extremely important from a policymaking level to understand this aspect because the overarching purpose of any CAP is to provide meaningful work to *everyone*. City-level policymakers across the United States need to understand the type of green jobs available in the market to create a clearer description of the workforce they need. This will enable each city to achieve feasible outcomes for actions and goals outlined in their CAPs. Second, we have interviewed only a few white-collar career professionals in universities and have not included trades or other workforce development professionals. This is a critical gap since the people that they interact with are starkly different than college graduates and it is just as important to include this analysis in the understanding of skills and roles that green jobs demand. Combining these interviews with an analysis of the political beliefs and value systems of the non-white collar^25^ workforce is also very important for policymakers in making clearer green job descriptions.

*This study gives an overview of the current state of green jobs in Worcester and provides insights beneficial to employers and job seekers alike. Though limited in scope, our analysis is critical in reducing implementation gaps.* As we learned from our interviews and the scraped data, employers and job seekers are often unsure as to what a green job is and whether the skills they have or need are “green,” or not. We found out that green jobs in Worcester are concentrated in terms of industry (energy, construction, waterworks) and siloed in terms of required skill and experience level, degree level, and distance from major population centers. It identifies that there is a need in cities like Worcester for a broadening and development of multiple pathways to a green job that will help the workforce get employed. By publishing these data, we hope to send employers a signal that they need to clearly understand and broaden their definition of what a green job is while trying to align it to GWP (or any other CAP) goals. Similarly, it also signals job seekers to broaden their search to different industries using different keywords to access jobs easily.

The study also raises broader conversations around “climate mainstreaming” and how it is lacking when it comes to CAP planning in cities. Although the term does not have a fixed definition (Brouwer et al., 2013), it is broadly understood as a practice that involves taking climate change planning into account at all levels “rather than as stand-alone measures or a separate sector” (Schaar, 2008, p. 1). Our study highlights that the GWP and the Worcester area still need more work in bringing climate change awareness to all spheres. For example, jobs in the trades (electricians or sanitation workers), which have already been in a decline for quite some time in the United States^26^ (Handel, 2024; Kalejaiye, 2023), are also often not referred to as green jobs in the job market despite contributing to sustainability sectors. It indicates that there is a lack of climate change conversations and thinking in trade unions and organized labor employment. However, this lack of thinking is prevalent in other employment too as most of the advertised jobs are “white-collar,” ignoring those without a formal college education or working in the trades. College graduates may not be educated about the benefits of a green job in the trades, which is exacerbated by the fact that these jobs are not advertised as “green jobs” in the first place. In a similar vein, job seekers in the trades may not see value in seeking “green jobs.”

Therefore, we suggest that the GWP (and other entities in Worcester) consider taking measures to promote climate-facing principles as an integral part of their planning apparatus and approach, policy documents, and outcomes. The decoupling of climate change considerations from the planning process also speaks to the siloed nature of climate planning processes and the fragmented conversations around climate change that leave out significant parts of the population at all levels. The causes for this “siloing” can be many (such as climate change communication^27^, disbelief in climate change, political will for messaging) and warrant further study. Overall, a broadening of the perspectives around climate change adaptation, the implemented measures to solve problems, and the green jobs involved would pave the way for a more inclusive and just adaptation in cities while also creating a multi-skilled workforce that can address multiple climate adaptation needs. The results also indicate the city might need to perform a climate mainstreaming assessment on the GWP implementation to understand where they currently stand (Gim and Shin, 2023).

We are expanding this pilot study both within Worcester and to a different Massachusetts city called New Bedford, replicating this process over a larger dataset with more types of jobs. Using the larger data set, we are planning on doing cluster analysis to understand where the jobs are in Worcester and New Bedford. We are also investigating other platforms; for example, how would the same analysis fare on government-used platforms like O*NET or on more transparent job platforms like Glassdoor? Moreover, we are also interviewing different types of career professionals, such as those who work as career professionals in trade unions or trade schools. As noted above, the discussants in this study are all career professionals where university students search for degree-related jobs. Urban Workforce or American Job Center offices, trade unions, and technical and vocational schools will provide access to a greater set of jobs and job seeker types that our current dataset cannot encompass. This wider job set will also help in generating more fine-tuned results through more sophisticated NLP algorithms that can also further evaluate the potential of machine learning in this field. The goal is to identify a suite of different factors, skills, roles, and responsibilities that either gate or allow access to green jobs in Massachusetts cities. Future scholars could expand upon this pilot study in other cities, domestic and international. The resources and methodologies used in this study are widely available and support a rich analysis. Through an amalgamation of the outlined techniques and other local contexts unique to a region, we believe that scholars can create a fabric of green job and workforce development perspectives that cities can use to achieve better outcomes in their respective climate adaptation plans.

## Data Availability

The raw data supporting the conclusions of this article will be made available by the authors, without undue reservation.

## Footnotes

^1^In addition to funding, planning, and other resources.

^2^For example, it could lead to situations such as a lack of certified workers or unemployment of a trained green workforce.

^3^See https://greenjobs.net/

^4^See https://climatebase.org/

^5^“Gateway Cities are midsize urban centers that anchor regional economies around the state. While Gateway Cities face stubborn social and economic challenges as a result, they retain many assets with unrealized potential. These include existing infrastructure and strong connections to transportation networks, museums, hospitals, universities and other major institutions, disproportionately young and underutilized workers, and perhaps above all, authentic urban fabric” (MassInc, n.d.).

^6^NLP enables “computers to understand and communicate with human language” (Stryker and Holdsworth, 2024).

^7^The Council’s Comprehensive Climate Action Plan is still in progress, (expected to complete in 2025) but initial reports have shown that stormwater management is being considered as a priority (Baltimore Metropolitan Council, 2024)

^8^See https://climatejobs.ai/

^9^We had an ethical concern around this method because scraping for research data collection is a relatively gray area. However, we confirmed that the WPI Institutional Review Board does not require any approval or authorization for it as long as we adhered to company policies. We also found that we were unclear about Indeed’s scraping policy, where the Terms of Service prohibit scraping but URL we used (indeed.com/jobs?) is not disallowed on the robots.txt file (a file that governs what can or cannot be accessed by automated programs).

^10^See https://www.parsehub.com/

^11^The Big Picture visions are Energy, Buildings and Transportation, Natural Systems, Social Equity, and Education and Awareness (City of Worcester, 2021, p. 14).

^12^See https://kumu.io/

^13^pandas is an analysis software in the python language which allows for powerful manipulations of large datasets with the help of arrays called dataframes.

^14^nltk is a toolkit that allows us to pre-process and perform analysis on natural language. See https://www.nltk.org/

^15^sklearn is a machine learning library for python. See https://scikit-learn.org/stable/getting_started.html

^16^See https://otter.ai/

^17^See https://climatechangejobs.com/, https://greenjobs.net/, https://greenjobs.greenjobsearch.org/

^18^To note, we had to input the keyword “solar” instead of “green jobs” to get this result.

^19^We also used the same search parameter of “within 50 miles of Worcester, MA” in the job search platform.

^20^See https://www.wgbh.org/news/local/2024-04-25/whats-faster-train-or-car-worcester-to-boston-commuters-say-its-not-that-simple-a-choice

^21^See https://data.census.gov/table/ACSST5Y2023.S2504?q=car%20ownership&g=160XX00US2582000

^22^See https://data.census.gov/table/ACSST5Y2023.S2504?q=car%20ownership&g=010XX00US_160XX00US2582000

^23^See https://www.census.gov/quickfacts/fact/table/worcestercitymassachusetts/AGE295223

^24^ *Indeed.com*

^25^Jobs like blue collar, pink collar, gray collar, etc.

^26^Because of high turnovers, lack of knowledge transfers and training programs, lack of political will, and beliefs in climate change.

^27^Our interviews reveal (see Table 1), there might also be something missing in the usual way the city has been communicating about the importance of green jobs to universities.
